# Modeling the Improved Visual Acuity Using Photodiode Based Retinal Implants Featuring Fractal Electrodes

**DOI:** 10.3389/fnins.2018.00277

**Published:** 2018-04-24

**Authors:** William J. Watterson, Rick D. Montgomery, Richard P. Taylor

**Affiliations:** Physics Department, University of Oregon, Eugene, OR, United States

**Keywords:** retinal implant, fractal, photodiode, neural prosthetic, irradiance safety limits

## Abstract

Electronically restoring vision to patients blinded by severe retinal degenerations is rapidly becoming a realizable feat through retinal implants. Upon receiving an implant, previously blind patients can now detect light, locate objects, and determine object motion direction. However, the restored visual acuity (VA) is still significantly below the legal blindness level (VA < 20/200). The goal of this research is to optimize the inner electrode geometry in photovoltaic subretinal implants in order to restore vision to a VA better than blindness level. We simulated neural stimulation by 20 μm subretinal photovoltaic implants featuring square or fractal inner electrodes by: (1) calculating the voltage generated on the inner electrode based on the amount of light entering the photodiode, (2) mapping how this voltage spreads throughout the extracellular space surrounding retinal bipolar neurons, and (3) determining if these extracellular voltages are sufficient for neural stimulation. By optimizing the fractal inner electrode geometry, we show that all neighboring neurons can be stimulated using an irradiance of 12 mW/mm^2^, while the optimized square only stimulates ~10% of these neurons at an equivalent irradiance. The 20 μm fractal electrode can thus theoretically restore VA up to 20/80, if other limiting factors common to retinal degenerations, such as glia scarring and rewiring of retinal circuits, could be reduced. For the optimized square to stimulate all neighboring neurons, the irradiance has to be increased by almost 300%, which is very near the maximum permissible exposure safety limit. This demonstration that fractal electrodes can stimulate targeted neurons for long periods using safe irradiance levels highlights the possibility for restoring vision to a VA better than the blindness level using photodiode-based retinal implants.

## 1. Introduction

The promise of restoring vision to patients blinded by dry age-related macular degeneration (AMD) and retinitis pigmentosa (RP) has spurred the development of retinal implants worldwide (Chow et al., 2004; Palanker et al., 2005; Shire et al., 2009; Zrenner et al., 2011; Humayun et al., 2012; Ayton et al., 2014; Stingl et al., 2015; Hornig et al., 2017). In the United States alone, an estimated ~50,000 people are blind (with visual acuity < 20/200) due to dry AMD (Congdon et al., 2004; Brightfocus Foundation, 2015) and ~20,000 due to RP (Grover et al., 1996). Central to both AMD and RP is the loss of the light-detecting photoreceptors (i.e., rods and cones), followed by a regressive remodeling of the remaining retinal neurons (Marc and Jones, 2003; Marc et al., 2003). This retinal remodeling involves a host of destructive processes such as rewiring of retinal circuits, neuronal migration, glia hypertrophy, and partial neuron death (Marc et al., 2003). In total though, the extent to which retinal remodeling prohibits bionic restoration of vision is not well understood. The hope is the surviving retina can detect patterned electrical stimulation, coherently transmit the signals to downstream visual areas, and perhaps plastically adapt to the stimulation over time. Currently, retinal implants restore vision up to a visual acuity of 20/1260 for epiretinal implants (positioned at the front of the retina) (Humayun et al., 2012) and 20/546 for subretinal implants (positioned at the back of the retina) (Zrenner et al., 2011; Stingl et al., 2015). However, the restored acuity for subretinal implants has only been achieved in one patient; 86% have no measureable restored acuity. Therefore, restoring vision beyond even the blindness level would represent a revolutionary breakthrough in retinal implant performance.

**Figure 1:**
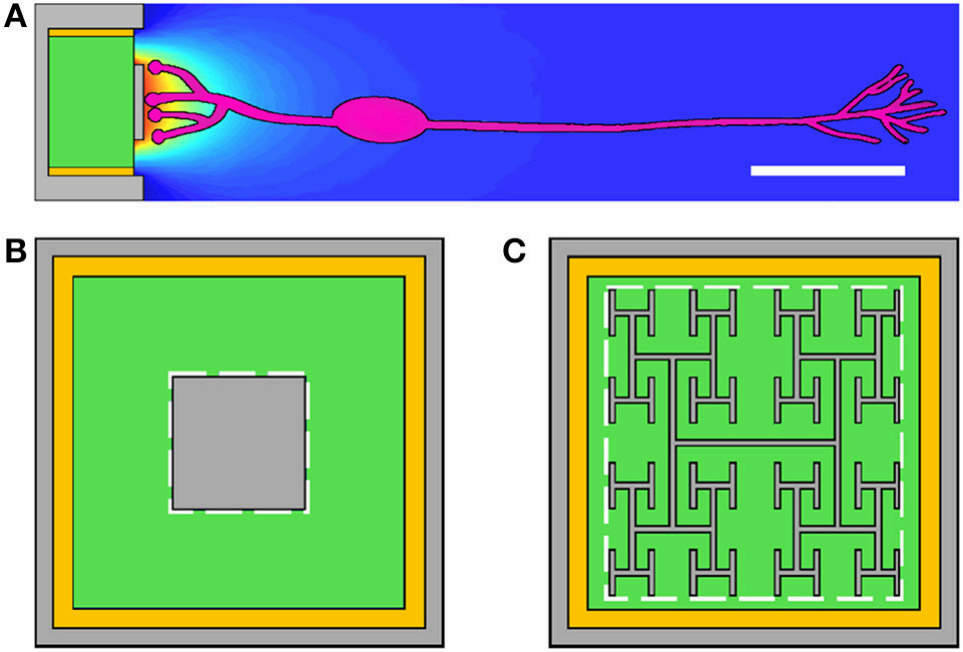
**(A)** The subretinal implant features a silicon photodiode (green), an inner electrode (gray), an outer grounded electrode (gray), and an insulating layer (yellow) between the photodiode's sides and the outer electrode. Current generated in the photodiode produces a voltage difference between the inner and outer electrodes which extracellularly stimulates bipolar neurons (pink). Scale bar is 20 μm. The conventional inner electrode is square shaped **(B)**, while we consider a fractal design based on a repeating H pattern **(C)**. The white dashed lines indicate the bounding perimeters of the inner electrodes.

Today's photodiode-based subretinal implants feature arrays of up to 1,500 photodiodes on 1–3 mm implants (Zrenner et al., 2011; Lorach et al., 2015). Each photodiode (“pixel”) is 70 μm wide. A prototypical design for a subretinal photodiode is shown in Figure 1A. Radiation incident on the silicon generates a voltage difference between an inner electrode and an outer grounded electrode. The associated electric field extends into the extracellular fluid of the retina and stimulates nearby bipolar neurons which then pass their signals downstream to ganglion neurons and from there to the visual cortex. Traditional designs employ a square-shaped inner electrode (Figure 1B). Proponents of the square electrode design face a predicament though; the electrode's surface area should be maximized to increase its electrical capacitance so that the field generated by the large amount of charge on the electrode extends far enough into the extracellular fluid to stimulate the neurons. Unfortunately, increasing the surface area also blocks more light from entering the underlying photodiode which reduces the inner electrode voltage and the associated electric field.

Recently, we proposed using fractal inner electrodes, featuring branched patterns that repeat at increasingly fine size scales (Figure 1C), as the ideal solution to this problem (Watterson et al., 2017). The sidewalls of the repeating branches generate a large surface area, and hence capacitance, while the gaps between the branches allow the light to pass through. We also proposed that this fractal design might offer additional favorable properties, including enhanced neural stimulation due to the close proximity of the neurons to the electrode (due to the fractal's surface texture promoting neural adhesion), favorable optical properties (including extraordinary transmission whereby the transmitted light intensity is greater than that expected from a simple pixel count of the photodiode's exposed area), and an increase in mechanical flexibility (which could be exploited to facilitate less obtrusive surgery and also to allow implants to conform to the curved surface at the back of the eye) (Watterson et al., 2017).

To quantify the impact of the fractal electrode's enhanced capacitance, we previously modeled a 20 μm-wide fractal and simulated the stimulation of retinal neurons when a bias, *V*, was applied to the electrode (Watterson et al., 2017). We found that all neighboring neurons (i.e., all neurons immediately above the electrode) were stimulated by 0.32 V for the fractal electrode while the equivalent square electrode required 0.9 V. Significantly, this fractal bias lies within the maximum voltage (i.e., the open-circuit voltage of 0.6 V) that a silicon photodiode can generate. Our result therefore highlighted the possibility that a single 20 μm-wide photodiode featuring a fractal inner electrode might be able to stimulate the retina while the square electrode design would require two or more photodiodes connected in series to accumulate the necessary voltage, with current designs using 3 photodiodes (Mathieson et al., 2012). The resulting order of magnitude decrease in pixel size for fractal-based retinal implants was predicted to have a crucial impact on visual acuity—the fractal implant has the potential to deliver 20/80 vision, which would allow restoration of vision to the level required for ambulatory tasks for the first time (Watterson et al., 2017).

However, a crucial question was left unanswered by this study. Although the required stimulation voltage is less than the photodiode's maximum possible voltage, what voltage does a 20 μm-wide photodiode featuring a fractal inner electrode actually generate under appropriate light intensities? Here we put the fractal implant proposal to the test by simulating the full operation of an implant's pixel (photodiode and electrode). Optimization of this operation requires balancing the effects of a number of potentially competing parameters, including light transmission, electrode impedance, electrode capacitance, and geometric factors influencing the electric field's penetration into the surrounding fluid. To quantify this optimization, we tune the fractal parameters of an “H-tree” electrode, in particular the scaling rate of the branches (as quantified by the fractal dimension, *D*) and the number of iterations of the repeating patterns, in order to quantify the degree to which fractal electrodes can generate superior neural stimulation to the square.

We show that the best square electrode requires 290% more radiation to stimulate all of the neighboring neurons compared to the best fractal design. This has the important implication that, although fractal-based implants will require supplemental infrared radiation to be beamed into the eye, the level of infrared is well within safety limits while the square-based implant operates just barely within this limit. Furthermore, we show that whereas the 20 μm fractal implant has the potential to deliver a maximum 20/80 visual acuity, the square suffers a dramatic decrease in perceived image quality due to stimulating 90% fewer neurons when operated at the same radiation levels as the fractal implant. We also discuss various strategies for reducing the irradiance requirements.

## 2. Methods

The retinal implant's operation is simulated in 3 separate stages in order to manage the computing power restraints. Firstly, photodiode simulations calculate the electrode voltages based on the incoming radiation intensity. These voltages then serve as the input parameters for the electrode simulations which model the associated electric field penetration into the fluid surrounding the neurons. Finally, neuron simulations then determine if these extracellular voltages are sufficient to stimulate the bipolar neurons and pass a signal downstream to the ganglion neurons. An oscillating electrode potential is employed for the second 2 stages of our simulations to overcome ionic screening by the fluid [in today's implants, this oscillation is realized by modulating the light entering the photodiode (Mathieson et al., 2012)]. We focus on a sine wave modulation due to its universal applicability (Watterson et al., 2017) and exclude the inter-pulse rest period used in today's implants (Tsai et al., 2009; Zrenner et al., 2011) since they can be included post simulation without impacting our conclusions (Watterson et al., 2017). All model parameters are listed in Table 1.

**Table 1:** List of model parameters and their associated values.

**Parameter**	**Value**	**References**
TiN resistivity	20e-6Ω cm	Pierson, 1996
TiN specific capacitance	2.5mF/cm^2^	Gabay et al., 2007
TiN charge transfer resistance	3e5Ω cm^2^	Franks et al., 2005
Retina resistivity	3,500Ω cm	Kasi et al., 2011
Neuron membrane capacitance	1.1μ F/cm^2^	Oltedal et al., 2009
Neuron cytoplasmic resistance	2.4e4Ω cm	Oltedal et al., 2009
Photodiode sheet resistance	20Ω /sq	Nelson, 2003
Photodiode-TiN contact resistance	2.4e-6Ω cm^2^	Sherman, 1990
Photodiode dark current density	1–1,000nA/cm^2^^*^	Wang et al., 2012
Photodiode responsitivity	0.30A/W	Wang et al., 2012

* *A photodiode dark current density of 100 nA/cm^2^ is a typical photodiode used in retinal implants today. Future implants could stimulate neurons more efficiently by minimizing the dark current. Varying values of dark current density from 1 to 1,000nA/cm^2^ are considered in section 4.2.1*.

### 2.1. Electrode construction

We consider single 20μm silicon photodiodes featuring an inner electrode (with either a square or fractal geometry) and an outer, grounded electrode (Figure 1). Both electrodes are 250 nm tall and are composed of titanium nitride (TiN), a commonly used retinal implant electrode material (Zrenner et al., 2011; Stingl et al., 2015). The silicon area (Figure 1, green) is 16 × 16μm and is surrounded by a 500 nm wide insulating layer (Figure 1, yellow). The bounding area (Figure 1, dashed white lines) for the square electrodes is varied between 50 and 200 μm^2^. The construction of the fractal electrodes is as follows.

**Figure 2:**
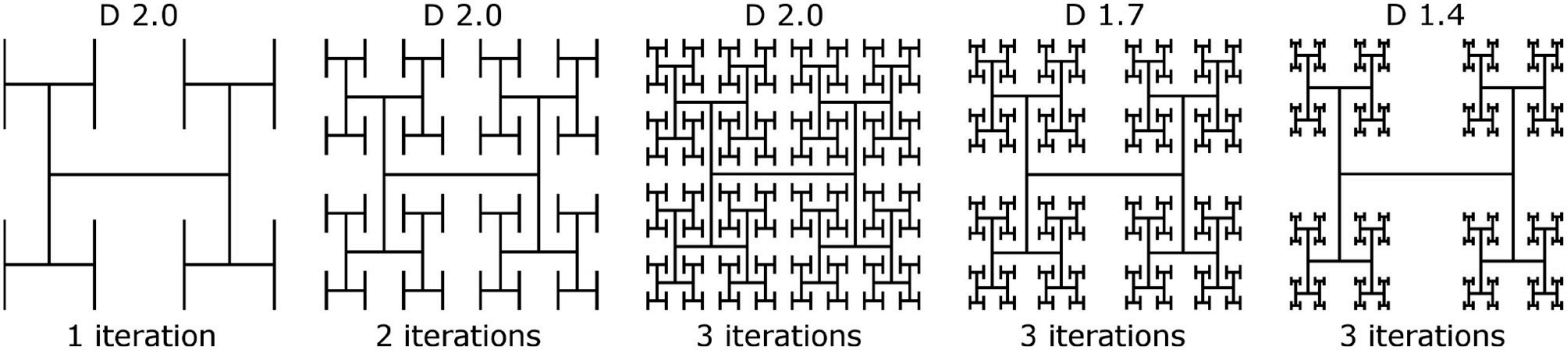
Construction of H-tree inner electrodes with increasing iterations from 1 to 3 at *D* = 2.0, and *D* values of 2.0, 1.7, and 1.4 at 3 iterations.

Mathematically exact fractals can be constructed by scaling an initial seed pattern and then iterating the scaled pattern toward increasingly fine size scales. The scaling rate, *L*, is set by the number of new patterns created, *N*, and *D*, according to the equation

(1) $$\begin{array}{r} N=L^{-D} \end{array}$$

where 1 ≤ *D* ≤ 2. Throughout this paper we model branched “H-tree” fractal electrodes. Figure 2 illustrates H-tree fractals which hold *D* fixed at 2.0 and increases the iterations from 1 to 2 to 3, and also H-trees which hold the iterations fixed at 3 and increases *D* from 1.4 to 1.7. In general, the H-tree electrode becomes more space filling for increasing iterations and increasing *D*. Each fractal electrode features line widths of 160 nm and a fixed bounding area of 15.4 × 15.2 μm. This line width was selected due to its ease of fabrication and also because it prevents the branches at different iterations from overlapping. In total, 13 electrode geometries were studied: 4 square electrodes with covering areas of 50, 100, 150, and 200 μm^2^ and 9 fractal electrodes from each combination of *D* values of 1.4, 1.7, and 2.0 and iterations of 1, 2, and 3. The covering area (i.e., the area of the top surface of each electrode) and total surface area (i.e., including the sidewall surfaces) of each electrode is given in Table 2.

**Table 2:** Covering area (i.e., area of the top surface of each electrode) and actual surface area (i.e., area including sidewalls) for each of the 13 electrode geometries.

**Electrode Geometry**	**Square**				**1 iteration**			**2 iterations**			**3 iterations**		
	**50 μm^2^**	**100 μm^2^**	**150 μm^2^**	**200 μm^2^**	***D* = 1.4**	***D* = 1.7**	***D* = 2.0**	***D* = 1.4**	***D* = 1.7**	***D* = 2.0**	***D* = 1.4**	***D* = 1.7**	***D* = 2.0**
Covering area (μm^2^)	50.0	100.0	150.0	200.0	13.3	13.8	14.2	21.9	25.5	28.4	34.0	44.4	55.1
Actual surface area (μm^2^)	57.1	110.0	162.2	214.1	55.1	57.1	58.7	90.4	105.3	117.2	140.4	183.1	227.4

### 2.2. Modified nodal analysis

**Figure 3:**
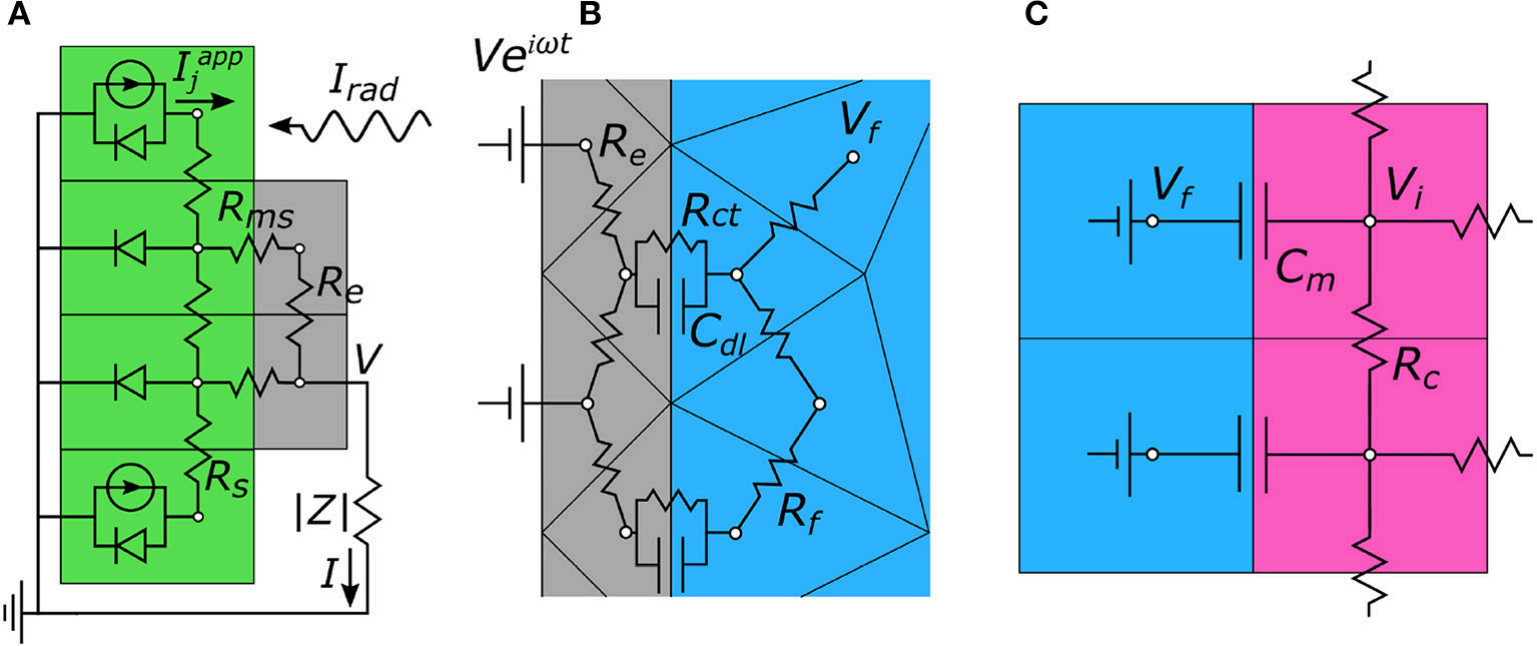
Two-dimensional representations of the equivalent circuit models used to calculate node voltages for each portion of the 3-step algorithm. **(A)** Step 1. The photodiode (green) generates a current, *I*^*app*^, in response to incoming radiation, *I*_*rad*_. This creates a voltage, *V*, on the electrode (gray) which then gets passed to step 2. **(B)** Step 2. *V* is applied to the electrode and the extracellular voltages, *V*_*f*_, in the electrolytic fluid (blue) are determined. **(C)** Step 3. The extracellular voltages are mapped onto the outside membrane of the model neurons (pink), and the neuron's internal voltage, *V*_*i*_, is calculated. In all, these simulations allow for the local change in neuron membrane potential, Δ*V*_*m*_ = Δ*V*_*i*_ − Δ*V*_*f*_, to be determined solely based of the intensity of incoming light, *I*_*rad*_.

The general strategy applied to the 3 simulation steps used to determine electrode, neuron, and photodiode responses is to mesh 3-dimensional geometries into a set of nodes, establish an equivalent circuit model between nodes (e.g., Figure 3 for 2-dimensional illustrations), and calculate the node voltages using modified nodal analysis (MNA) (Ho et al., 1975). Briefly, MNA determines node voltages by applying Kirchhoff's current conservation rule at each node along with the appropriate boundary conditions. For *n* node voltages, $\vec{V}\;$ = (*V*_1_, …, *V*_*n*_), and *m* applied voltage sources, $\vec{V}\;$^*app*^ = $\;\left(V_{1}^{app},\ldots ,V_{m}^{app}\right)$, the MNA system of equations is given by

(2) $$\left(\begin{array}{cc} G & A^{T}\\ A & 0 \end{array}\right)\left(\begin{array}{c} \vec{V}\\ \vec{I} \end{array}\right)\;=\;\left(\begin{array}{c} {\vec{I}}^{app}\\ {\vec{V}}^{app} \end{array}\right)$$

where *G* is an *n* × *n* matrix containing conductance elements between nodes, *A* is an *m* × *n* matrix that sets boundary conditions to the applied voltages and only contains zeros and ones, $\vec{I}\;$= (*I*_1_, …, *I*_*m*_), gives the *m* currents flowing through the applied voltage sources, and $\vec{I}\;$^*app*^ = $\;\left(I_{1}^{app},\ldots ,I_{m}^{app}\right)$ applies current sources to the *n* nodes. The lower right *m* × *m* matrix is zero. The system of equations is solved using the package SuperLU (Demmel, 1999; Li and Demmel, 2003).

### 2.3. Photodiode operation

The MNA algorithm outlined above is used to characterize the current and voltage generated by each photodiode under illumination. The photodiode is first recreated as a 2-layer cubic mesh featuring TiN electrode nodes in the top layer and semiconducting silicon nodes in the bottom layer (Figure 3A). The node-to-node impedances feature an electrode resistance between metal nodes, a sheet resistance between semiconducting nodes, and a contact resistance between metal and semiconducting nodes. The equivalent circuit model also includes the load impedance magnitude, |*Z*|, between the inner and outer electrodes (see section 2.4 and Equation 5). Under illumination, the photodiode current is modeled as an array of current sources (i.e., photocurrents generated from the incident radiation) in parallel with diodes (i.e., “dark” currents—thermal recombination between the electron and hole charge carriers that occurs even in the absence of light) (Figure 3A). Photocurrents are included only for nodes which are exposed to the radiation (i.e., not blocked by the inner electrode). The net current for node *j*, $I_{j}^{app}$, is given by an ideal diode under illumination according to

(3) $$\begin{array}{r} I_{j}^{app}\;=I_{sc}-I_{dark}\;=a\left(I_{rad}R-J_{0}\left(e^{V_{j}/V_{T}}-1\right)\right) \end{array}$$

where *a* is the node's top surface area, *I*_*sc*_ is the short-circuit current (current when |*Z*| = 0), *I*_*dark*_ the “dark” reverse current, *I*_*rad*_ the irradiance (radiant power per unit area), *R* the photodiode responsivity (amps generated per incoming watt of radiation), and *J*_0_ the dark current density at 0 V. The dark current density is estimated to be 100 nA/cm^2^ by comparing to similar microphotodiode subretinal implants (Chow et al., 2004; Wang et al., 2012). The thermal voltage, *V*_*T*_ = 0.0268 V, is the value at the body's temperature of 310 K. *V*_*j*_ is the voltage at node *j*. Semiconducting nodes below the top-contact only feature a dark current. The only quantity inserted in $\vec{V}\;$^*app*^ (Equation 2) is setting the ground potential to 0 V. For each photodiode, the MNA equation is solved iteratively using a global Newton method (Bank and Rose, 1981; Ceric, 2005) to determine the node voltages, $\vec{V}$, and the current flowing through the load impedance, *I*. We note that each electrode exhibited a maximum variation in metal node voltages of less than 1.3%. Each inner electrode is therefore approximately equipotential, and is measured by a single voltage at the central metal node, termed *V*. The relationship between *I* and *V*, gives the photodiode's *IV* curve.

In addition to the *IV* curves, one common characterization of subretinal implant photodiodes that occurs on the laboratory benchtop, prior to implantation, is to measure the open-circuit voltage, *V*_*oc*_, by leaving the connection between the inner and outer electrodes open (i.e., infinite |*Z*|). For a given irradiance, *V*_*oc*_ only depends on the photodiode parameters and the inner electrode geometry. Here, the open circuit voltage, *V*_*oc*_, can be estimated by

(4) $$\begin{array}{r} V_{oc}\;=V_{T}\ln\left(\frac{I_{rad}RA_{pd}}{J_{0}A_{tot}}+1\right) \end{array}$$

where *A*_*tot*_ is the total photodiode area and *A*_*pd*_ is the photodiode area not blocked by the electrode.

### 2.4. Electrode operation

We previously described how the MNA algorithm outlined in section 2.2 is used for calculating the extracellular voltages generated by the electrodes (see Watterson et al., 2017 for a detailed explanation). Briefly, a 1 mm^3^ cubic domain containing the inner electrode, the outer grounded electrode, and the extracellular space is meshed into a set of tetrahedral nodes. Next, an equivalent circuit model is created which defines the node-to-node impedances. The fluid-fluid nodes are resistive (*R*_*f*_), while the fluid-electrode nodes feature a capacitor (*C*_*dl*_) and resistor (*R*_*ct*_) in parallel, which model charge screening and reversible oxidation-reduction reactions at the electrode, respectively (Figure 3B) (Merrill et al., 2005). The fluid resistivity is taken to be 3,500 Ω cm (Kasi et al., 2011). The applied voltage boundary conditions, $\vec{V}\;$^*app*^ in Equation (2) are set as follows. An oscillating voltage, *V*_*e*_ =*Ve*^2π*ift*^, (where the value of *V* is inputted from the photodiode simulations) is applied to the inner electrode while the outer electrode is held at 0 V. The remaining boundary conditions are set to be insulating for the plane in which the electrode is located, and 0 V at the other 5 faces of the cubic domain. There are no applied current sources in this portion of the simulations so $\vec{I}\;$^*app*^ = $\;\vec{0}$ (Equation 2). Having established the equivalent circuit model along with the boundary conditions, Equation (2) can be solved for the *n* complex valued node voltages and *m* complex currents through the boundary condition nodes. The voltage in the electrolytic fluid outside the neuron is termed *V*_*f*_. The load impedance magnitude, |*Z*|, (which is set by the network of *R*_*f*_, *C*_*dl*_, and *R*_*ct*_ components) can also be calculated by

(5) $$\begin{array}{r} \left|Z|\;=\;|V|/|I\right| \end{array}$$

where |*I*| is the current leaving the inner electrode. Additionally, we calculate the charge density, *Q*_*ph*_, at each node delivered on the electrode surface per positive phase of voltage by

(6) $$\begin{array}{r} Q_{ph}\;=\;\int_{0}^{\frac{1}{2f}}dtC_{dl}|\frac{d\left(V_{e}-V_{f}\right)}{dt}|\;=\;2C_{dl}|V_{e}-V_{f}| \end{array}$$

### 2.5. Neuron stimulation

The neuron stimulation is described in detail elsewhere (Watterson et al., 2017). Briefly, the extracellular voltages, *V*_*f*_, calculated in section 2.4 induce a change in the membrane potential, Δ*V*_*m*_ = Δ*V*_*i*_ − Δ*V*_*f*_, in the bipolar neurons located near the electrode, where Δ*V*_*i*_ is the change in internal potential of the neuron and Δ*V*_*f*_ is the change in electrolytic fluid potential. In turn, these bipolar neurons pass their signal downstream to retinal ganglion cells when Δ*V*_*m*_ reaches a minimum of 15 mV at the bipolar neuron's soma (Yang and Wu, 1997).

Our model bipolar neurons are 100 μm long with a 10 μm soma centered 30 μm above the electrode surface, and dendrites which are just above the electrode's top surface (Wassle et al., 2009; Masland, 2012). In our simulations, each neuron features a cubic mesh. Passive rod bipolar neurons are quantified by a membrane capacitance of 1.1 μF/cm^2^ in parallel with a membrane resistance of 2.4 × 104 Ωcm^2^, along with an internal cytoplasmic resistivity of 130 Ω cm (Oltedal et al., 2009). For the applied stimulation frequencies used here (1 kHz), the resistive impedance is more than 2 orders of magnitude higher than the capacitive impedance. We therefore ignore the resistive component and create an equivalent circuit model containing solely membrane capacitances and internal cytoplasmic resistances (Figure 3C). The real and imaginary parts of the extracellular voltages obtained in section 2.4 are mapped onto the outside of the neuron's membrane and serve as a set of applied voltage sources, $\vec{V}\;$^*app*^ in Equation (2). The MNA equation is then solved to obtain the neuron's internal voltage at each node.

## 3. Results

### 3.1. Photodiode performance

**Figure 4:**
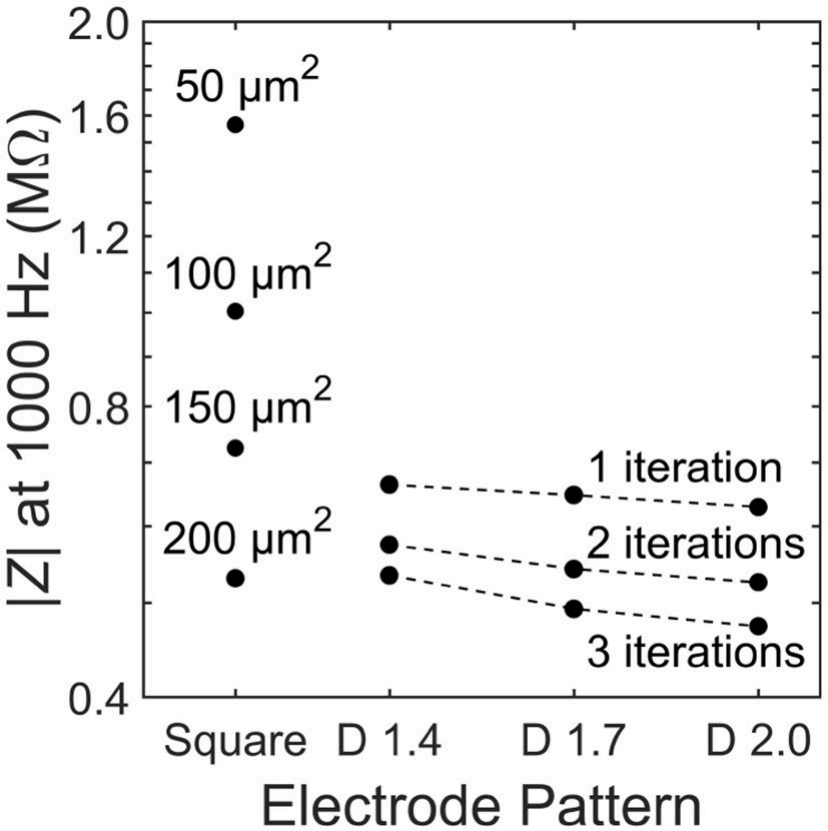
Load impedance, |*Z*|, for each of the 13 electrode geometries. Dashed lines depict constant iteration with varying D for fractal electrodes.

We first consider the load impedance, |*Z*|, because this will determine how close the photodiode operates at to open or closed-circuit. The load impedance for the square electrode is found to decrease with increasing electrode size (Figure 4). This is expected because the geometric contribution to the load impedance is inversely proportional to the inner electrode's effective surface area and directly proportional to the distance between the inner and outer electrodes. The fractal electrode reduces its impedance relative to the square by increasing its effective surface area (by maximizing the surface area via the large number of branch sidewalls) and decreasing the separation between inner and outer electrodes. This leads to a general trend of decreasing impedance for increasing *D* value and increasing iterations (Figure 4).

**Figure 5:**
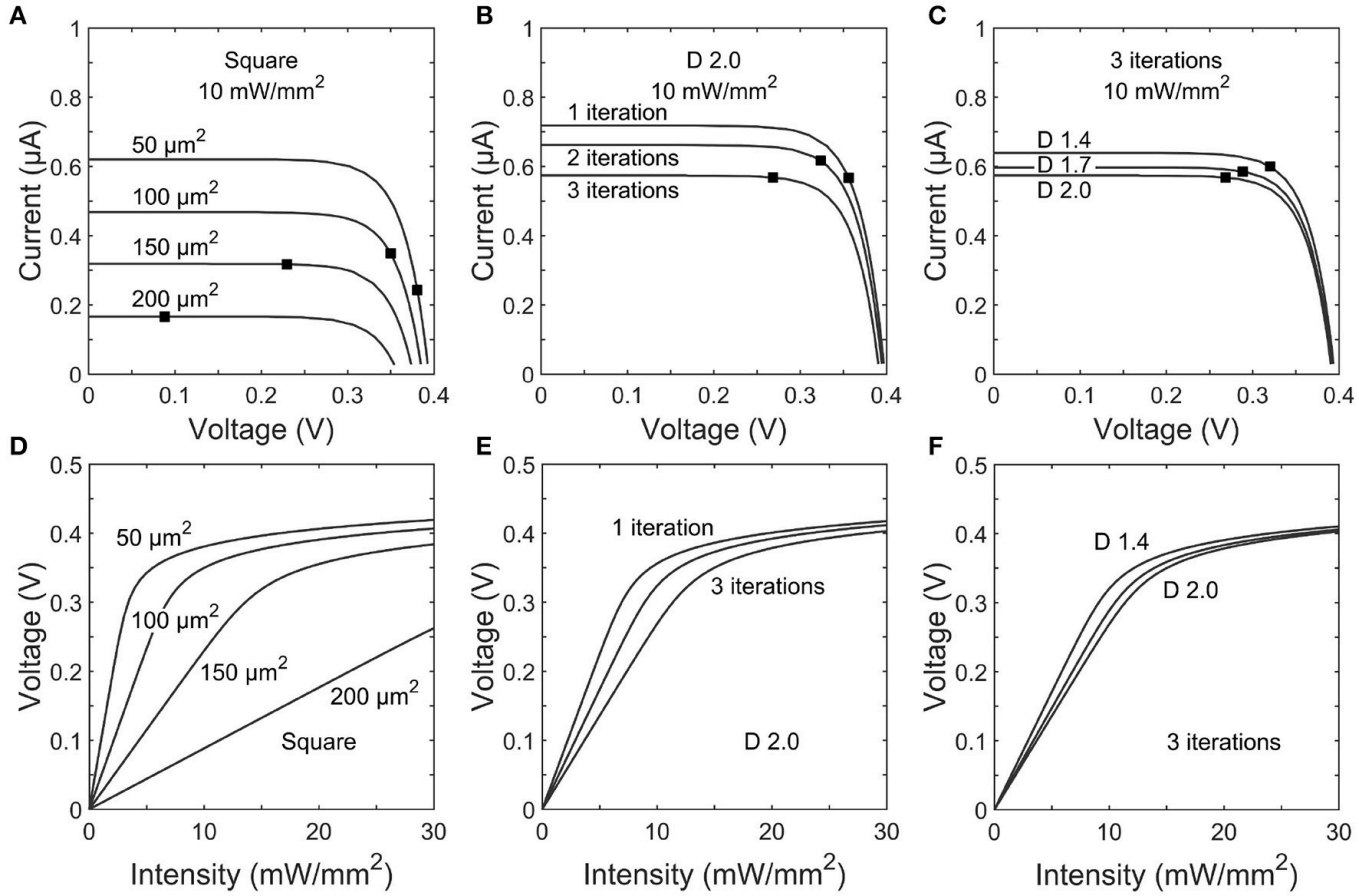
Top row: *IV* curves for photodiodes with **(A)** square, **(B)** 1–3 iteration *D* = 2.0 H-trees, and **(C)** *D* = 1.4, 1.7, or 2.0 H-trees with 3 iterations inner electrode geometries. The black dot on each trace indicates the load impedance for that electrode operating at 1 kHz stimulation frequency. Bottom row: Voltage generated for varying incident irradiance on **(D)** square, **(E)** 1–3 iteration *D* = 2.0 H-trees, and **(F)** *D* = 1.4, 1.7, or 2.0 H-trees with 3 iterations inner electrode geometries.

Figures 5A–C show the *IV* curves for photodiodes with square and fractal electrode geometries under an illumination of *I*_*rad*_ = 10 mW/mm^2^. Each electrode exhibits an open-circuit voltage of ~0.40 V and a short-circuit current proportional to the exposed photodiode area. In a conventional solar cell, the load impedance would be chosen to maximize the power generated. However, here each photodiode has a load impedance set by the electrode geometry. The black dot on each trace in Figures 5A–C shows the operating point on the *IV* curve set by the impedances reported in Figure 4.

The voltages generated by each electrode geometry as a function of irradiance display several common characteristics (Figures 5D–F). Firstly, at low voltages the slope of each trace is given by Δ*V*/Δ*I*_*rad*_ =*R*|*Z*|*A*_*pd*_, where *A*_*pd*_ is the unblocked photodiode area. The electrodes with smaller covering areas have both large |*Z*| and *A*_*pd*_ and therefore generate relatively high voltages at the lower intensities. Secondly, as the voltage begins to approach the open circuit voltage, increasing illumination intensity provides minimal increases in the electrode voltage.

### 3.2. Extracellular fields and neural stimulation

**Figure 6:**
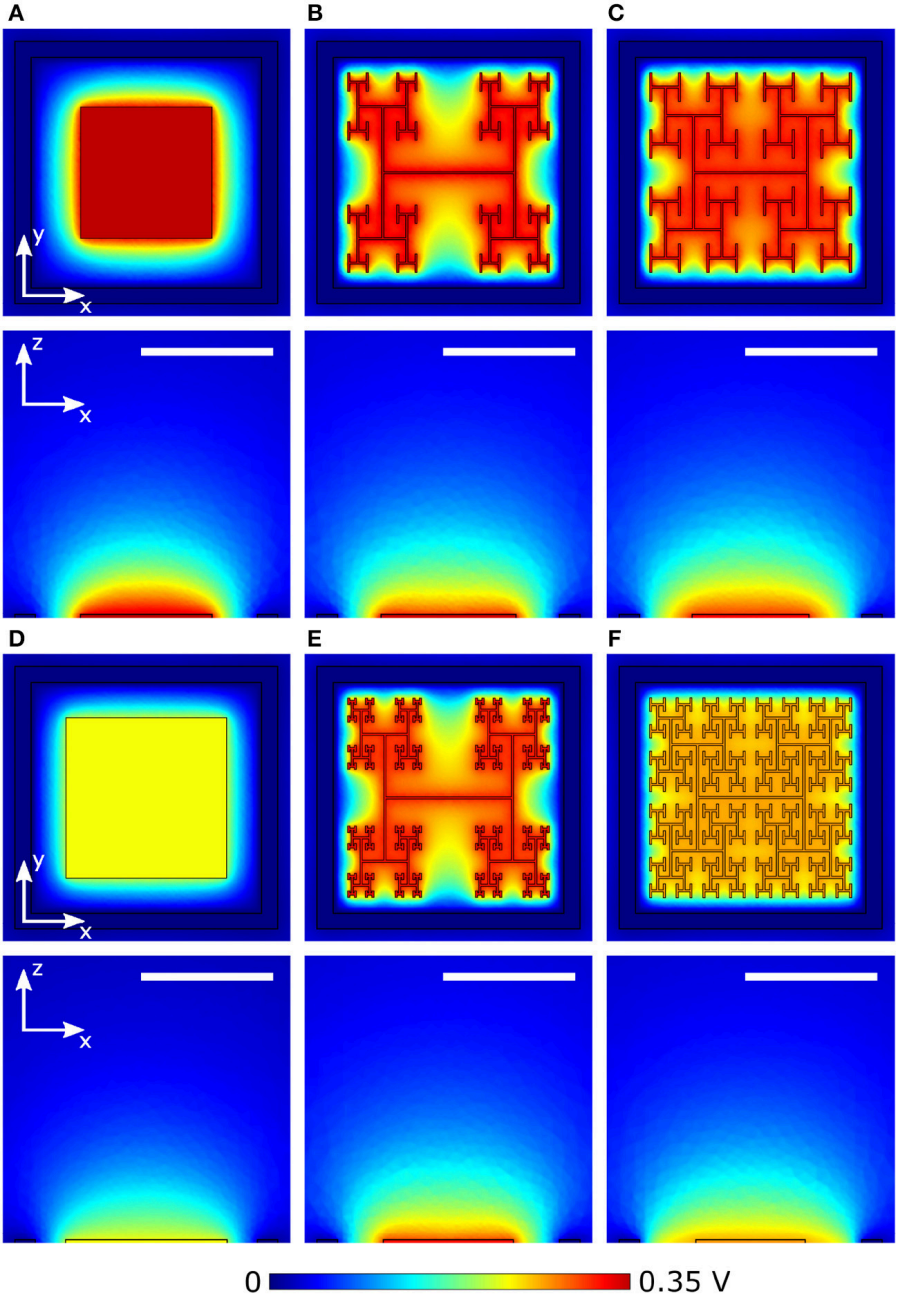
Magnitude of extracellular voltages under 10 mW/mm^2^ incident irradiance at 1 kHz stimulating frequency delivered by square electrodes of size **(A)** 100 μm^2^ and **(D)** 150 μm^2^, and *D* = 1.4 H-trees with **(B)** 2 and **(E)** 3 iterations, and *D* = 2.0 H-trees with **(C)** 2 and **(F)** 3 iterations. Rows 1 and 3 show horizontal slices at the top surface of the inner electrode, while rows 2 and 4 show vertical slices through the center of the electrode. The white scale bars are all 10 μm.

The results of section 3.1 highlight the importance of electrode geometry when determining the voltage generated for a given illumination. However, electrode geometry also influences how the field from this voltage extends into the extracellular liquid and this can lead to competing considerations. For instance, Figures 6A,D show the effect of increasing a square electrode's area from 100 to 150 μm^2^. As expected from the decrease in |*Z*| and *A*_*pd*_, the larger electrode's voltage decreases significantly and the field does not therefore extend as far vertically into the liquid as the smaller electrode's field. However, the field from the larger electrode has the advantage of extending further horizontally within the pixel. An inevitable consequence of the square design therefore is that fields that extend far vertically do not extend far horizontally and vice versa.

The fractal design offers a potential solution for optimizing this competition. The fractal electrode generates high voltages for a given illumination (Figure 5). Furthermore, its maximal capacitance (due to the large surface area generated by the branch sidewalls) allows a large amount of charge to reside on the electrode and this generates a large field for a given applied voltage, which will penetrate far vertically into the liquid. Because the electrode spreads further laterally than a square electrode for the same covering area, the fractal electrode's field will also extend far horizontally within the confined area of a single photodiode. We note that the horizontal spread does not significantly spread into the neighboring pixel due to the outer ground, and therefore the electrical crosstalk remains minimal (see Watterson et al., 2017). However, the presence of the gaps in the fractal design needs to be taken into account. Figures 6B,E show the fields for the *D* = 1.4 electrode; both the 2 and 3 iteration electrodes feature large gaps which reduce the extracellular voltage in the central region. For the *D* = 2.0 fractals shown in Figures 6C,F, increasing the number of iterations from 2 to 3 reduces the voltage but the field spreads out relatively uniformly across the entire pixel. Given that larger extracellular fields generally induce large depolarizations, Δ*V*_*m*_, of the bipolar neurons, it is clear from the above that careful geometric optimization will be required to supply a large voltage which extends into the most extracellular space.

**Figure 7:**
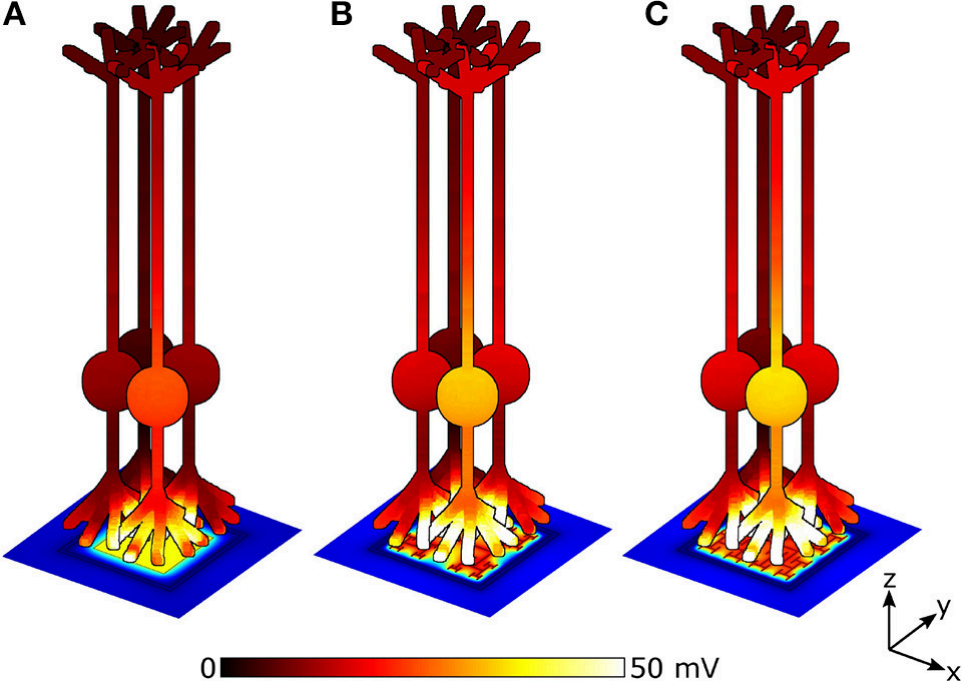
Peak membrane depolarizations achieved during a voltage oscillation for a patch of 4 bipolar neurons above the **(A)** 150 μm^2^ square, **(B)** 2 iteration *D* = 1.4 fractal, and **(C)** 2 iteration *D* = 2.0 fractal electrodes. The front most neuron in each image is centered above the pixel. Bipolar neurons are 100 μm tall and images are drawn to scale. The remaining 5 of the 9 neighboring neurons are not shown for clarity.

The stimulation efficiency for each design is determined by measuring Δ*V*_*m*_, for a patch of 9 bipolar neurons directly above each electrode. Figure 7 depicts Δ*V*_*m*_ for a patch of 4 of the 9 neighboring bipolar neurons above electrodes under equivalent illuminations of 10 mW/mm^2^. Because the 150 μm^2^ square electrode (Figure 7A) blocks a larger percentage of the underlying photodiode and therefore has a lower voltage on the inner electrode, the neurons above the square depolarize less compared to the 2 iteration *D* = 1.4 and *D* = 2.0 H-trees (Figures 7B,C). Additionally, the fractal electrode's *D* value influences the field distribution in the extracellular space, leading to varying neural depolarizations. For instance, although the voltage on the 2 iteration *D* = 1.4 H-tree is slightly larger than the voltage on the 2 iteration *D* = 2.0 H-tree (0.38 vs. 0.36 V), the depolarizations are larger for neurons above the 2 iteration *D* = 2.0 H-tree (Figures 7B,C).

**Figure 8:**
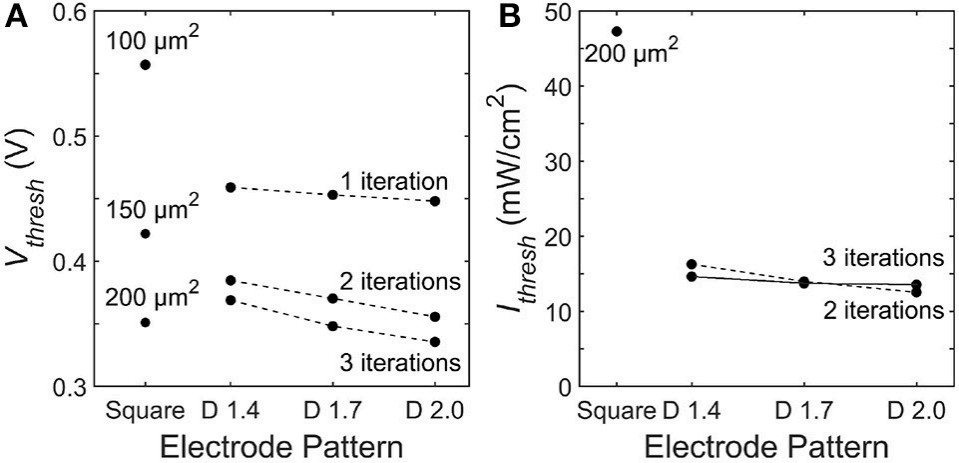
Threshold **(A)** electrode voltages, *V*_*thresh*_, and **(B)** irradiant intensities, *I*_*thresh*_, necessary to induce Δ*V*_*m*_ = 15 mV somatic depolarization in all 9 bipolar neurons above each electrode. The dashed line connects 2 iteration fractals and the solid line connects the 3 iteration fractals.

To quantify the stimulation efficiency, we define the electrode threshold stimulating voltage, *V*_*thresh*_, as the electrode voltage at which all 9 neighboring bipolar neurons reach a somatic depolarization of Δ*V*_*m*_ = 15 mV. Previous experiments show this 15 mV condition results in stimulation of the downstream ganglion neurons Yang and Wu (1997). For square electrodes, increasing the electrode area reduces *V*_*thresh*_ due to an increase in capacitance. Likewise, increasing the capacitance for fractal electrodes either by increasing the number of iterations or increasing the *D* value leads a to lower *V*_*thresh*_ (Figure 8A). However, as discussed in section 3.1, increasing the electrode's covering area also reduces the voltage generated on the inner electrode. Therefore, efficient stimulation requires a careful optimization of supplying enough voltage from the photodiode and maintaining a low *V*_*thresh*_.

Across all of the electrode patterns, the 2 iteration *D* = 2.0 fractal provides the best balance between these 2 competing factors (Figure 8). In particular, the incident radiation required to stimulate all neighboring bipolar neurons is 290% more for the best square electrode of 200 μm^2^ than for the the 2 iteration *D* = 2.0 fractal. We note that at their threshold voltages, the maximum surface charge density, *Q*_*ph*_, of the optimized electrodes are *Q*_*ph*_ = 0.67 mC/cm^2^ for the 200 μ m^2^ square electrode and *Q*_*ph*_ = 0.93 mC/cm^2^ for the 2 iteration *D* = 2.0 H-tree. These charge densities are less than the 1 mC/cm^2^ safety limit for TiN electrodes based on the charge densities that induce hydrolysis (Weiland et al., 2002).

### 3.3. Stimulation frequency

So far, we have considered stimulating pulses operating at a frequency of 1 kHz. However, conventional subretinal implants being developed today use stimulating frequencies ranging from 250 Hz to 2 kHz (Zrenner et al., 2011; Mathieson et al., 2012; Lorach et al., 2015). In order to verify the fractal maintains a lower threshold irradiance at lower stimulating frequencies than 1 kHz, we repeated the above analysis for the 150 μm^2^ square, the 200 μm^2^ square, and the 2 iteration *D* = 2.0 fractal at a stimulating frequency of 250 Hz.

**Figure 9:**
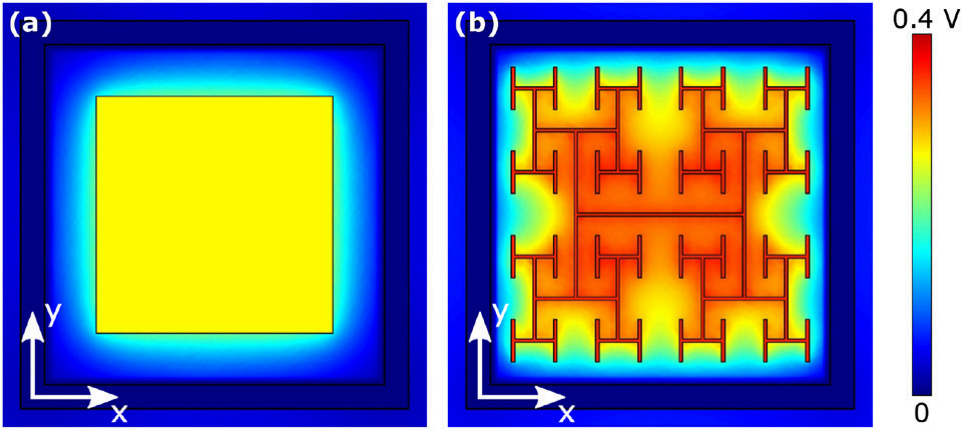
Magnitude of extracellular voltages under 10 mW/mm^2^ incident irradiance at 250 Hz stimulating frequency delivered by **(A)** 150 μm^2^ square and **(B)** 2 iteration *D* = 2.0 H-tree electrodes.

First, lowering the stimulating frequency causes a rise in the load impedance, |*Z*|, for each geometry due to an increase in capacitive impedance at the electrode-electrolyte interface. This increased |*Z*| leads to a larger voltage generated on the inner electrode (i.e., the operating point on the *IV* curve shifts to a higher voltage). For example, under 10 mW/mm^2^ illumination, reducing the frequency from 1 kHz to 250 Hz causes an increase in the inner electrode voltage from 0.09 to 0.11 V for the 200 μm^2^ square and from 0.34 to 0.36 V for the 2 iteration *D* = 2.0 fractal. Simultaneously though, the increased impedance leads to a smaller spreading in the extracellular field generated by each electrode (Figure 9). Additionally, the lower frequency causes smaller depolarizations in the bipolar neurons due to a higher capacitive membrane impedance.

Combining all of these factors, we find the threshold irradiances, *I*_*thresh*_, necessary to depolarize all 9 surrounding neurons at 250 Hz are 90 mW/mm^2^ for the 150 μm^2^ square, 42 mW/mm^2^ for the 200 μm^2^ square, and 15 mW/mm^2^ for the 2 iteration *D* = 2.0 fractal. Therefore, lowering the stimulating frequency from 1 to 250 kHz causes a reduction in *I*_*thresh*_ for the 200 μm^2^ square and an increase in *I*_*thresh*_ for the 2 iteration *D* = 2.0 fractal. However, the fractal implant still requires 64% less irradiance intensity to stimulate all surrounding neurons than the best square design.

## 4. Discussion

### 4.1. Fractal advantages

#### 4.1.1. Irradiance efficiencies

We have shown that the threshold irradiance for the best square design is 290% higher than the best optimized fractal. Specifically, for a 20 μm pixel with typical photodiode and electrode properties found in today's retinal implants, that *I*_*thresh*_ for the optimized fractal, 2 iteration *D* = 2.0 H-tree, is 12 mW/mm^2^ while for the best square, 200 μm^2^, *I*_*thresh*_ is 47 mW/mm^2^ when operated at *f* = 1 kHz. For reference, the irradiance of direct sunlight at the Earth's surface is 1 mW/mm^2^.

Because today's implants, including our fractal designs, require more radiation than that supplied by direct sunlight, pulses of infrared (IR) radiation are repeatedly beamed into implants (Mathieson et al., 2012; Lorach et al., 2015). For 20 μm pixels, the square design therefore requires 290% more power beamed in than the same sized pixel featuring our fractal design. An alternative approach to reducing power requirements is to increase pixel size to collect more radiation. For example, some conventional implants beam in 4 mW/mm^2^ to 70 μm pixels (Mathieson et al., 2012). However, increases in pixel size reduce visual acuity (see section 4.1.3).

#### 4.1.2. Intensity safety limits

The light intensities which can be safely beamed into the eye without overheating the retina are set by the maximum permissible exposure limits (Delori et al., 2007). These intensities are labeled for single pulses of light as $I_{safety}^{sp}$, and for pulses which repeat indefinitely at some given frequency, $I_{safety}^{av}$. In today's implants, pulses of infrared (IR) radiation are repeatedly beamed into the implant (Mathieson et al., 2012; Lorach et al., 2015). IR is used because the cornea and lens are transparent to IR, the silicon photodiode responsivity is maximal in the IR, and the maximum permissible exposure limits are higher for IR than for visible. Assuming IR light of an identical wavelength to that used in today's implants is beamed into the square and fractal photodiodes considered in this paper, then $I_{safety}^{sp}\;$= 285*f*^0.25^ and $I_{safety}^{av}$ = 5.2 mW/mm^2^ (Mathieson et al., 2012). For single pulses of frequency *f* = 1 kHz, the optimized fractal electrode (2 iteration *D* = 2.0) is a factor of 24 below $I_{safety}^{sp}$ while the best square (200 μm^2^) is only a factor of 6 below.

For repeated stimulation by sinusoidal pulses as considered here, the average threshold intensity is $I_{thresh}^{av}\;$=$\;\frac{F}{\pi f}I_{thresh}$ where *F* is the interpulse frequency. Current implants operate at an interpulse frequency up to *F* = 20 Hz (Zrenner et al., 2011; Lorach et al., 2015). However, since the critical flicker-fusion rate (the rate at which 95% of people cannot perceive an image as flickering) is 80 Hz (Myers, 2003), future implants could aim to operate at a higher frequency of *F* = 80 Hz. At *f* = 250 Hz and *F* = 80 Hz, $I_{thresh}^{av}$ = 4.3 mW/mm^2^ for the 200 μ m^2^ square and $I_{thresh}^{av}$ = 1.5 mW/mm^2^ for the 2 iteration *D* = 2.0 H-tree. While both the square and fractal have $I_{thresh}^{av}<I_{safety}^{av}$, the square is quite close to surpassing the safety limit. The reduction in threshold intensity afforded by the 2 iteration *D* = 2.0 H-tree therefore ensures a long-term safe operation of the implant.

Finally, we note that the above results are based on subretinal stimulation of a healthy retina in which bipolar depolarizations of Δ*V*_*m*_ = 15 mV enable the signal to be passed to downstream ganglion neurons (Yang and Wu, 1997). However, *in vitro* experiments using 400 μm diameter platinum electrodes placed behind the rods and cones found eliciting a ganglion spike required a mean threshold stimulating current of 3.6 times more for a degenerate mouse retina model of RP vs. a healthy retina (Jensen and Rizzo, 2008). Because *V*_*thresh*_ for the 20 μm square and fractal electrodes lies beyond the linear region of the *V* vs. *I*_*rad*_ graphs (Figures 5D–F), increasing *I*_*rad*_ will only provide a small increase in *V*. Therefore, the 20 μm squares are unlikely to reach the threshold stimulation condition for the degenerate retina without surpassing the intensity safety limits. However, because increasing the photodiode size has been shown to decrease *I*_*thresh*_ in healthy retinas (Wang et al., 2012), larger square sizes should result in linear increases in *V* necessary for the stimulation of a degenerate retina. We note that the fractal advantages outlined in sections 4.1.1 and 4.1.2, and in particular those related to an increased capacitance, will reduce the stimulation condition for both healthy and degenerate retinas and thus mitigate the need for significantly larger electrodes. Future experiments will measure the precise irradiance required for stimulation of healthy and degenerate retinas from fractal electrodes.

#### 4.1.3. Visual acuity

The fractal inner electrode incorporated into a 20 μm pixel is capable of stimulating all the surrounding bipolar neurons within the maximum permissible exposure safety limits. The visual acuity associated with a 20 μm pixel is calculated as follows. Visual acuity is inversely related to the number of arcminutes at which an object can be resolved. In natural vision, 20/20 acuity equates to resolving two lines separated by 1 arcmin, corresponding to a 5 μm pixel at the retina. For electronically restored vision with a 20 μm pixel, as considered here, the maximum restored acuity is therefore 4 times reduced from 20/20 vision, corresponding to 20/80 vision. We note that when operated at the same illumination level of 12 mW/mm^2^ as the fractal electrode, the best square design only stimulates 1 of the 9 neurons above the 20 μm pixel. This reduced stimulation will generate fewer spiking events per second in the downstream ganglion neurons, which will reduce the perceived image quality (Stett et al., 2000).

In reality, the restored acuity will be worse than the upper limit of 20/80 due to a number of factors including electrical crosstalk (whereby the voltage on one electrode pixel stimulates neurons above neighboring pixels) (Watterson et al., 2017), glia scarring (Polikov et al., 2005), stimulation of passing axon fibers (Beyeler et al., 2017), surgical complications (Ghodasra et al., 2016), and remodeling of the retina after photoreceptor loss (Marc et al., 2003). However, we have previously demonstrated that electrical crosstalk for a 20 μm fractal electrode does not stimulate the neurons above a neighboring pixel (Watterson et al., 2017) and we expect the fractal electrode will reduce glia scarring since glia scarring is reduced on textured surfaces (Butterwick et al., 2009; Piret et al., 2015). Unwanted stimulation of passing axon fibers for our implant should remain low, because while epiretinal stimulation of passing ganglion axon fibers can significantly distort the perceived image (Beyeler et al., 2017), the majority of patients receiving subretinal implants report percepts as round spots, with only a subset seeing arc-like visual percepts indicative of unwanted stimulation of passing axon fibers (Wilke et al., 2011).

Perhaps the most critical barrier to success for retinal implants is remodeling of the inner retina after photoreceptor loss (Marc et al., 2003). This retinal remodeling encompasses a wide range of destructive processes such as rewiring of retinal circuits, neuronal migration, glia hypertrophy, and neuron death, among others. However, the net effect of retinal remodeling on subretinal implant performance is not well understood and could potentially be mitigated by nutritional enrichment to the retina during the early stages of disease onset (Barone et al., 2012) and/or early intervention with a retinal implant during RP or AMD progression (Marc et al., 2003). The fractal electrode's increased surface texture and mechanical flexibility could potentially improve neural proximity to the electrode and reduce the harmful effects of retinal remodeling. In total, any negative factors which limit restored acuity will apply to both the square and the fractal. However, the fractal's improved stimulation, coupled with the possibility of reduced glia scarring and increased neuronal adhesion, indicate the fractal design will lead to better patient outcomes in restored acuity.

### 4.2. Further refinements to fractal-based implants

#### 4.2.1. Photodiode parameters

**Figure 10:**
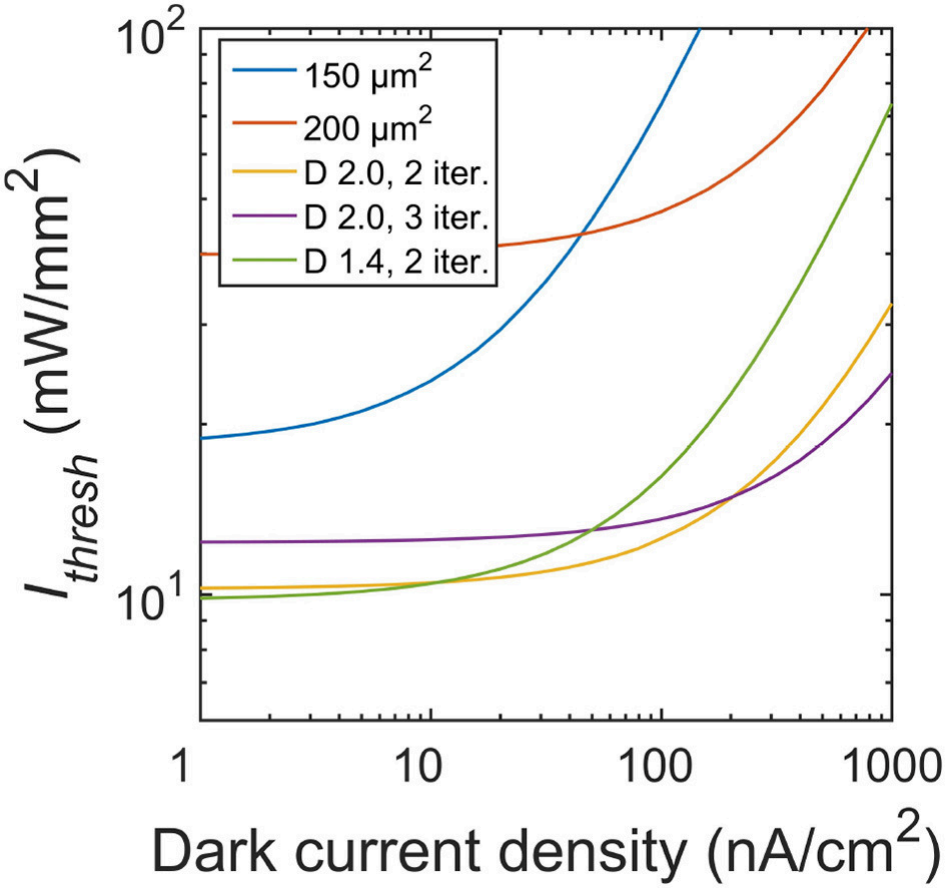
Threshold irradiances for varying dark current densities. Photodiodes used in subretinal implants today operate at a dark current density of 100 nA/cm^2^ (equating to *V*_*oc*_ ~ 0.4 V). At this *J*_0_, the *D* = 2.0 H-tree with 2 iterations is the optimal geometry. Reducing the dark current density to 1 nA/cm^2^ (*V*_*oc*_ ~ 0.5 V) would lead to the *D* = 1.4 H-tree with 2 iterations being the optimal geometry.

In this study, we focused on the silicon microphotodiodes used in today's retinal implants. Higher performing photodiodes will inevitably reduce the irradiance requirements. For example, we modeled the silicon photodiode using an open-circuit voltage, *V*_*oc*_ ~ 0.4 V (Chow et al., 2004; Wang et al., 2012). However, in principle the *V*_*oc*_ silicon voltage can reach as high as 0.6 V. A higher *V*_*oc*_ leads to a larger range of irradiance in which Δ*V*/Δ*I*_*rad*_ is linear (Figure 5), thereby reducing the required intensity necessary to induce the 15 mV depolarization in neighboring neurons. For instance, at a larger *V*_*oc*_ ~ 0.5 V (corresponding to a dark current density *J*_0_ ~ 1 nA/cm^2^), the *I*_*thresh*_ for the optimal fractal electrode drops to 10 mW/mm^2^ compared to 12 mW/mm^2^ for *V*_*oc*_ ~ 0.4 V (Figure 10). Interestingly though, the 2 iteration *D* = 1.4 geometry now corresponds to the lowest threshold irradiance as compared to the 2 iteration *D* = 2.0 at *V*_*oc*_ ~ 0.4 V (*J*_0_ ~ 100 nA/cm^2^).

The threshold irradiance can also be reduced by increasing the photodiode's responsivity. The responsivity, *R*, is given by

(7) $$\begin{array}{r} R=QE\frac{\lambda }{1240}\left[\text{A}/\left(\text{W}\cdot \text{nm}\right)\right] \end{array}$$

where *QE* is the quantum efficiency (bounded between 0 and 1) and λ is the wavelength in nm. The responsivity can thus be improved by either increasing *QE* or increasing λ. However, the maximum λ which will induce a photocurrent is determined by the inverse of the bandgap energy of the photodiode material. For silicon, λ is typically limited to ~1,000 nm. Other III-V semiconductors, such as InGaAs, feature lower bandgaps and thus a higher sensitivity to longer infrared wavelengths. For example, commercially available InGaAs photodiodes typically feature a responsivity of ~1 A/W at an absorption wavelength of ~1,600 nm. This responsivity of ~1 A/W for an InGaAs photodiode is approximately 3 times higher than the silicon photodiodes modeled in this study, and would translate to a factor of 3 reduction in the threshold irradiance for both the square and fractal electrodes. However, incorporating a material such as InGaAs into a subretinal implant would require careful passivation schemes since the arsenic is cytotoxic (Bausch et al., 2013). Alternatively, we also note the responsivity can be increased by etching fractal holes into the silicon (Fazio et al., 2016).

Subretinal implants being developed today use an electrode made from either titanium nitride (TiN) (Zrenner et al., 2011; Stingl et al., 2015) (as modeled here) or iridium oxide (IrOx) (Lorach et al., 2015). Since TiN and IrOx feature similar charge injection limits and interfacial electrode impedances (Weiland et al., 2002), we anticipate the fractal electrode composed of IrOx would still significantly outperform the square electrode.

#### 4.2.2. Electrode transparency

Transparency of the inner electrode is critical to the implant operation. As a first step, the current study considered a simplified ‘pixel count’ model of light transmission into the silicon. This pixel model is based on ray optics, in which light either reflects off the electrode surface or passes through the gaps. This pixel model is valid when *b* ≫ λ, where *b* is the gap size and λ is the wavelength. In reality, because the electrode features gap sizes which are either the same order of magnitude as the wavelength of light (*b* ~ λ) or smaller (*b* < λ), one of two different optical regimes will dominate. In the diffraction regime, where *b* ≪ λ, the subwavelength gaps transmit light by a reduced factor proportional to (*b*/λ)^2^ of that predicted from ray optics (Bethe, 1944). This leads to more space filling fractals (i.e., smaller effective gap sizes) transmitting less radiation into the photodiode. In the surface plasmon regime, when *b* ~ λ, fractals have been shown to exhibit extraordinary transmission of light, i.e., the radiation entering the photodiode is greater than that predicted from a simple pixel count (Matteo and Hesselink, 2005; Li et al., 2013; Afshinmanesh et al., 2014). For example, by appropriately selecting the number of iterations for a given fractal, the transmission efficiency through fractal apertures at resonant wavelengths can be increased by over an order of magnitude compared to square apertures (Matteo and Hesselink, 2005). The most obvious fractal pattern for maximizing extraordinary transmission is the Hilbert fractal, featuring just one gap size which could be matched to the resonance condition (Afshinmanesh et al., 2014). However, the Hilbert's dimension is set to *D* = 2.0 and so this type of fractal would lack the ability to tune the electrical stimulation parameters described in this paper, along with other favorable properties such as cell adhesion (Gentile et al., 2013) and charge injection capacity (Park et al., 2018). Accordingly, we predict that the H-tree will remain the optimal fractal design, and that by tuning its *D* value and the number of iterations to emphasize the plasmonic effect, the intensity could be maximized and the transmitted wavelength (i.e., color) could be tuned (Bao et al., 2008; Gottheim et al., 2015)

#### 4.2.3. Outer electrode design

**Figure 11:**
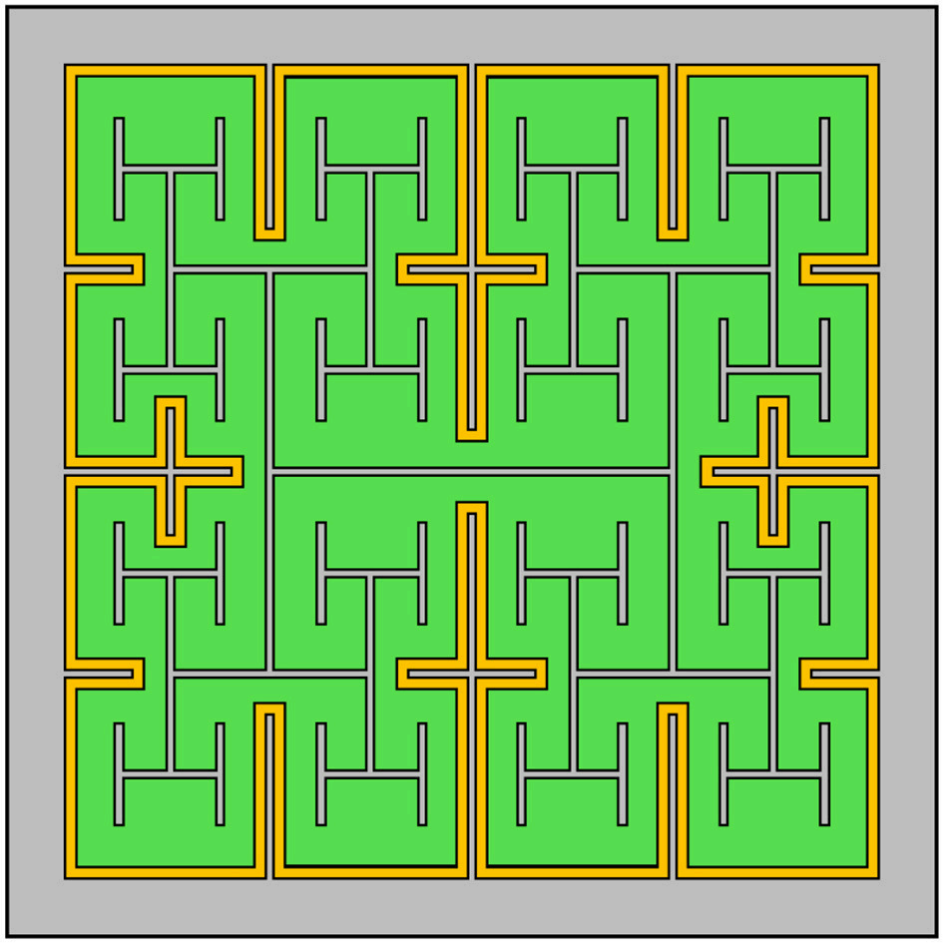
Design of a subretinal implant featuring a fractal H-tree inner electrode and a grounded outer fractal electrode (in this case, one iteration of a cross pattern). The photodiode is depicted in green, the two electrodes in gray and the insulator in yellow.

Given the advantages gained by adopting a fractal design for the inner electrode, it is natural to consider the impact of including an outer fractal electrode (Figure 11). The fractal ground design significantly increases capacitance by reducing the distance between the inner and outer electrodes (Samavati et al., 1998). However, this increase in capacitance leads to stronger in-plane electric fields, whereas efficient bipolar neuron stimulation requires stronger electric fields perpendicular to the electrode's surface. Therefore, solely increasing capacitance may not directly translate into more efficient stimulation. We note, however, that strong in-plane variations in the electric field could translate to more efficient stimulation for implants designed to interface with peripheral nerves in the human arm (Golestanirad et al., 2013). Furthermore, for retinal implants, the fractal ground also reduces the amount of exposed photodiode area, resulting in less current generated per watt of inputted radiation. Finally, the 20 μm fractal electrodes without a fractal ground already featured a maximum surface charge density, *Q*_*ph*_, near the 1 mC/cm^2^ safety limit for TiN electrodes. Reducing the distance between the inner and outer electrodes will further increase the surface charge density, potentially resulting in *Q*_*ph*_ exceeding the safety limit. Combining all of these considerations, we hypothesize that including a fractal ground electrode would negatively impact neural stimulation from 20 μm photodiode-based implants.

## 5. Conclusions

Branched fractal electrodes best balance a number of competing requirements necessary for efficient neural stimulation from photodiode implants. (1) The gaps between the branches transmit large amounts of light into the underlying photodiode, thereby generating high electrode voltages. (2) The sidewalls of the branches create a large surface area and therefore a high electrode capacitance. For a given voltage, the fractal electrode then holds a large amount of charge and the electric field generated by this charge extends vertically far into the extracellular space. (3) The gaps ensure that, for a given covering area, the fractal has a large bounding area. By carefully selecting the optimal *D* and number of iterations, the field penetrates the gaps and ensures a uniform field that extends far laterally. Combined, the above factors ensure a large uniform field that penetrates a sufficient volume of extracellular space to maximize neural stimulation.

Consequently, the 20 μm fractal implant stimulates all of the surrounding bipolar neurons using 74% less irradiance compared to the square. In addition to an improved efficiency, the fractal's decreased threshold irradiance holds important consequences for the safe operation of future implants. For long-term continuous operation of implants, the square is just barely within the maximum permissible exposure limit while the fractal is significantly within. Moreover, for equivalent irradiance of 12 mW/mm^2^ illuminating the best optimized square and fractals, the fractal stimulates ~90% more neurons. Thus, whereas the 20 μm fractal implant has the potential to deliver a maximum of 20/80 vision acuity, the square suffers a significant decrease in perceived image quality. When the performance factors reported here are coupled with potentially beneficial adhesive and mechanical properties, it is clear that fractal electrodes have the potential to dramatically improve the restored visual acuity from subretinal implants.

## Author contributions

WW, RM, and RT designed the study. WW and RM performed the analysis. WW and RT drafted the manuscript.

### Conflict of interest statement

The authors declare that the research was conducted in the absence of any commercial or financial relationships that could be construed as a potential conflict of interest.
